# Association between *MTHFR* gene C677T polymorphism and gestational diabetes mellitus in Chinese population: a meta-analysis

**DOI:** 10.3389/fendo.2023.1273218

**Published:** 2023-10-30

**Authors:** Xi Tan, Hongqin Chen

**Affiliations:** Department of Obstetrics and Gynecology, West China Second University Hospital, Sichuan University, Key Laboratory of Birth Defects and Related Diseases of Women and Children (Sichuan University) of Ministry of Education, Chengdu, Sichuan, China

**Keywords:** methylenetetrahydrofolate reductase, C677T polymorphism, gestational diabetes mellitus, China, meta-analysis, trial sequential analysis

## Abstract

**Background and purpose:**

The relationship of the methylenetetrahydrofolate reductase (*MTHFR*) gene C677T polymorphism with the incidence of gestational diabetes mellitus (GDM) in the Chinese population remains controversial. This study aimed to further clarify the effect of the *MTHFR* gene C677T polymorphism on GDM risk among Chinese pregnant women based on current evidence.

**Methods:**

Several databases were searched up to July 29, 2023 for relevant case-control studies. The numbers of patients with and without the T allele of the *MTHFR* gene C677T polymorphism in the GDM and control groups were determined, and all statistical analyses were performed by RevMan 5.3 software and STATA 15.0 software. Trial sequential analysis (TSA) was performed by TSA version 0.9 beta software to determine the required information size.

**Results:**

A total of 17 case-control studies involving 12345 Chinese participants were included. The pooled results demonstrated that the T allele of the *MTHFR* gene C677T polymorphism was significantly associated with an increased risk of GDM, which was manifested by the five gene models of the *MTHFR* C677T polymorphism [T vs. C: odds ratio (OR)=1.59, P=0.03; TT vs. CC: OR=2.24, P<0.001; TC vs. CC: OR=1.28, P=0.05; (TT+TC) vs. CC: OR=1.55, P=0.003; TT vs. (TC+CC): OR=1.89, P<0.001]. Subgroup analysis based on the regions indicated that the significant relationship between the T allele of the *MTHFR* gene C677T polymorphism and an increased risk of GDM was detected only among the southern population [T vs. C: OR=1.62, P=0.09; TT vs. CC: OR=2.22, P=0.004; TC vs. CC: OR=1.17, P=0.28; (TT+TC) vs. CC: OR=1.43, P=0.03; TT vs. (TC+CC): OR=1.97, P=0.006]. TSA plots showed that the information sizes for the association between the *MTHFR* gene C677T polymorphism and GDM risk were sufficient in the homozygote (TT vs. CC) and recessive (TT vs. TC+CC) models.

**Conclusion:**

The *MTHFR* gene C677T polymorphism is closely related to susceptibility to GDM in the southern Chinese population, and the C-T mutation serves as an important genetic risk factor for GDM. More well-designed large case-control studies are needed to further confirm the above findings.

## Introduction

Gestational diabetes mellitus (GDM) is a common complication during pregnancy, posing significant health risks to both the fetus and the mother. The incidence of GDM has been rising, with reported rates ranging from 1% to 14% internationally and from 2% to 18% in China (1–4). Additionally, the onset of GDM is occurring at increasingly younger ages (2). GDM can have adverse effects on both the mother and the fetus, leading to complications such as miscarriage, preterm birth, diabetic ketoacidosis, infection, postpartum hemorrhage, fetal distress, macrosomia, neonatal respiratory distress syndrome, and neonatal hypoglycemia (5). Furthermore, both pregnant women with GSM and their offspring have an increased risk of developing type 2 diabetes in the long term (5). Therefore, identifying potential and modifiable risk factors for GDM is a valuable strategy to enhance the health status of pregnant women and their offspring.

Homocysteine (Hcy) is a reactive amino acid associated with vascular injury that impacts glucose and lipid metabolism. Previous studies have indicated that Hcy levels are higher in GDM patients than in women with normal pregnancies, suggesting a potential close relationship between Hcy levels and GDM and its complications (6). Relevant studies suggest that insulin resistance is a central factor in the development of GDM, and plasma Hcy levels are positively correlated with fasting insulin levels (7). The insulin resistance index directly influences plasma Hcy levels, although this remains a subject of significant debate (8, 9).

Methylenetetrahydrofolate reductase (*MTHFR*) can catalyze 5,10-methylenetetrahydrofolate to 5-methyltetrahydrofolate, participating in the remethylation of Hcy and keeping Hcy levels relatively low in the body. Variations in the *MTHFR* gene can lead to disruptions in multiple physiological and biochemical reactions in the body, such as modifications in the methylation of nucleic acids and proteins and the regulation of the cell cycle. The *MTHFR* gene is located on the short arm of human chromosome 1 (1p36.3), and polymorphisms in the *MTHFR* gene can result in reduced enzyme activity, causing folate metabolism disturbances, reduced 5-methyltetrahydrofolate production, impaired DNA methylation, metabolic disruptions of Hcy, and the accumulation of Hcy in the bloodstream. Currently, hundreds of single nucleotide polymorphisms (SNPs) have been identified in the *MTHFR* gene, with the C677T site being the most closely associated with diseases. When this gene polymorphism occurs, it may lead to reduced or impaired *MTHFR* enzyme activity, disrupted folate metabolism, and elevated Hcy levels and consequently result in related complications (10–13). Some studies have demonstrated that the *MTHFR* gene C677T polymorphism is closely related to the risk of diabetes mellitus (DM) (14, 15). Some investigators also revealed that the C-T mutation in the *MTHFR* C677T gene might lead to increased susceptibility to GDM (16, 17). However, whether the *MTHFR* gene C677T polymorphism leads to an increased risk of GDM in the Chinese population remains controversial.

Therefore, this meta-analysis aimed to further identify the effect of the *MTHFR* gene C677T polymorphism on GDM risk among Chinese pregnant women based on current evidence.

## Materials and methods

The current meta-analysis was performed according to the Preferred Reporting Items for Systematic Review and Meta-Analyses 2020 guidelines (18).

### Literature search

In this meta-analysis, the CNKI, VIP, WanFang, Medline, Web of Science and EMBASE databases were searched from inception to July 29, 2023. The following terms were used during the search: single nucleotide polymorphism, SNP, polymorphism, gestational diabetes mellitus, pregnancy-induced diabetes, methylenetetrahydrofolate reductase and *MTHFR*. The detailed search strategy was as follows: (single nucleotide polymorphism OR SNP OR polymorphism) AND (gestational diabetes mellitus OR pregnancy-induced diabetes) AND (methylenetetrahydrofolate reductase OR *MTHFR*). Moreover, MeSH terms and free-text words were both applied, and references cited in the included studies were also reviewed.

### Inclusion criteria

Studies that met the following criteria were included: 1) studies of the association between the *MTHFR* gene C677T polymorphism and the incidence of GDM among pregnant Chinese women; 2) studies in which the numbers of patients with and without the T allele of the *MTHFR* gene C677T polymorphism in the GDM and control groups were provided for the calculation of odds ratios (ORs) with 95% confidence intervals (CIs); and 3) high-quality studies with a Newcastle-Ottawa Scale (NOS) score of 6 or higher (19).

### Exclusion criteria

Studies that met the following criteria were excluded: 1) letters, editorials, animal trials, reviews or case reports; and 2) studies with duplicated or overlapped data.

### Data collection

The following information was extracted from each included study: the first author, publication year, sample size, number of patients with and without GDM, genotype detection method, number of patients with different genotypes and NOS score.

### Quality assessment

The NOS was applied to assess the methodological quality of the included studies because all included studies were cohort studies. As mentioned above, only studies with an NOS score ≥ 6 were included.

The literature search, literature selection, data extraction and quality assessment were independently conducted by two authors, and all disagreements were resolved by team discussion.

### Statistical analysis

The heterogeneity between studies was assessed by using I^2^ statistics and the Q test. If significant heterogeneity was detected, defined as I^2^ > 50% and/or P < 0.1, the random effects model was applied; otherwise, the fixed effects model was applied (20). ORs and 95% CIs were combined to evaluate the effect of this polymorphism on GDM risk under the allele model (T vs. C), homozygote model (TT vs. CC), heterozygote model (TC vs. CC), dominant model (TT+TC vs. CC) and recessive model (TT vs. TC+CC). The overall population was stratified according to region (northern vs. southern), and subgroup analysis was conducted by region. Sensitivity analysis was conducted to determine the sources of heterogeneity and assess the stability of the overall results. In addition, Begg’s funnel plot and Egger’s test were conducted to detect publication bias, and significant publication bias was defined as P < 0.05 (21, 22). If obvious publication bias was detected, then the fill-and-trim method was applied to identify potentially unpublished studies (23). The above analysis was performed by RevMan 5.3 software and STATA 15.0 software.

Trial sequential analysis (TSA) was conducted according to the guidelines of former publications (24, 25). A significance level of 5% for type I error and a significance level of 20% for type II error were set to evaluate the required sample size and TSA monitoring boundaries, which was performed by TSA version 0.9 beta software.

## Results

### Literature search and selection

**Figure 1:**
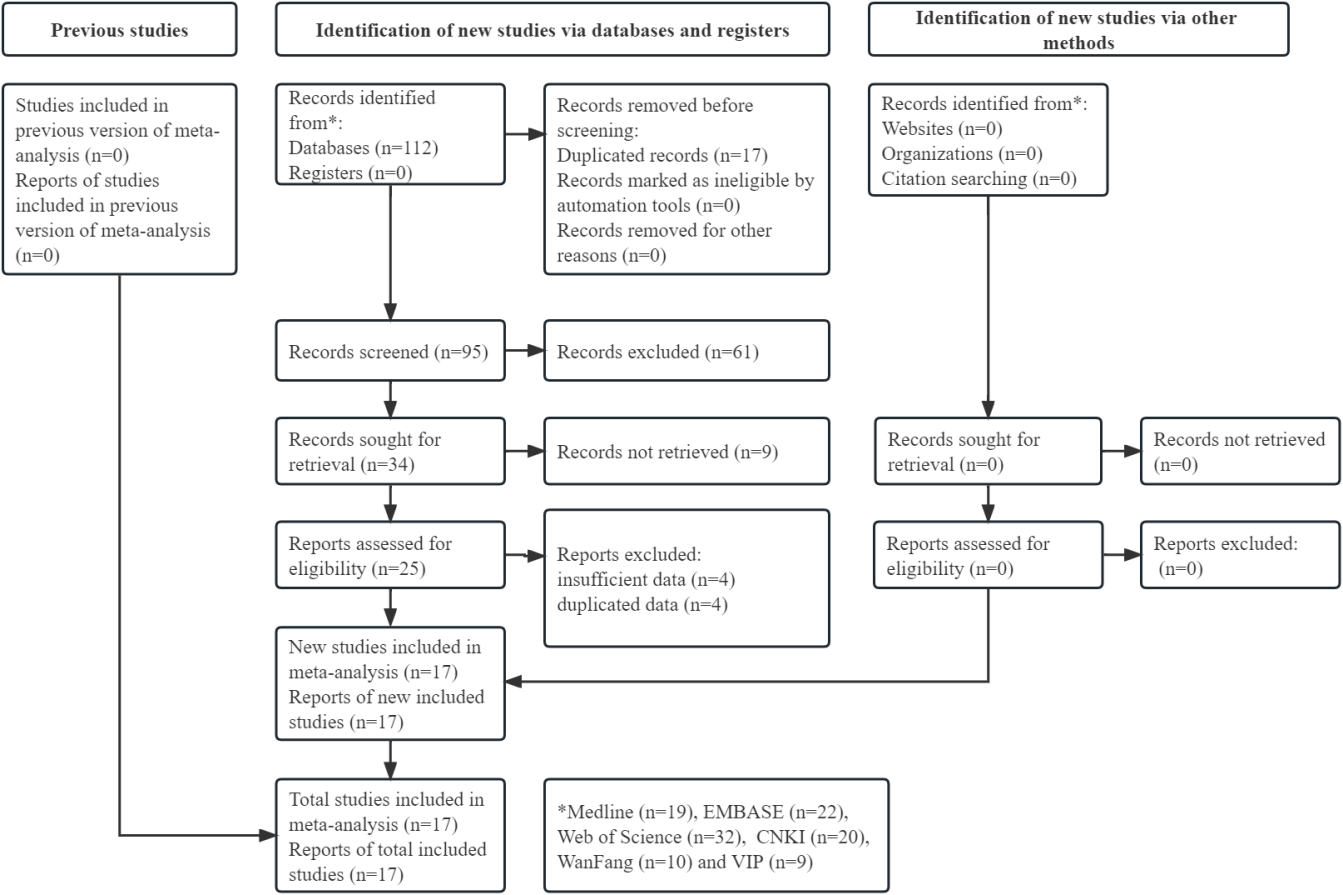
Prisma flow diagram of this meta-analysis.

The specific literature search and selection process is shown in **Figure 1**. Initially, 112 records were identified from the six databases. Ultimately, a total of 18 available studies were included in this meta-analysis after reviewing the titles, abstracts and full texts (14, 26–41).

### Basic characteristics of the included studies

As shown in **Table 1**, 12345 Chinese participants were enrolled, with sample sizes ranging from 60 to 5614, and nine studies were from southern China. Among the enrolled patients, 3439 were diagnosed with GDM. Detailed data are presented in **Table 1**. Specific information about the NOS score is shown in **Supplementary Table 1**.

**Table 1:** Basic characteristics of included studies.

Author	Year	Region	Sample size	GDM	Non-GDM	Genotyping method	Allele model				Homozygote model				Heterozygote model				Dominant model				Recessive model				NOS
							T-GDM	C-GDM	T	C	TT-GDM	CC-GDM	TT	CC	TC-GDM	CC-GDM	TC	CC	TT+TC-GDM	CC-GDM	TT+TC	CC	TT-GDM	TC+CC-GDM	TT	TC+CC	
Cheng (26)	2016	S	214	91	123	QF-PCR	50	132	56	190	10	51	3	70	30	51	50	70	40	51	53	70	10	81	3	120	7
Yang (27)	2016	S	363	166	197	TaqMan	96	234	151	241	16	85	30	75	64	85	91	75	80	85	121	75	16	149	30	166	7
Guan (28)	2018	N	60	27	33	Sequencing	24	30	31	35	6	9	13	15	12	9	5	15	18	9	18	15	6	21	13	20	6
Li (30)	2019	N	490	158	332	Sequencing	81	77	132	200	45	44	54	122	69	44	156	122	114	44	210	122	45	113	54	278	8
Xing (31)	2019	S	490	158	332	Sequencing	159	157	264	400	45	44	54	122	69	44	156	122	114	44	210	122	45	113	54	278	8
Chen (29)	2019	S	314	155	159	Sequencing	11	155	9	159	31	40	20	84	84	40	55	84	115	40	75	84	31	124	20	139	7
Ni (32)	2020	S	199	97	102	FQ-PCR	98	96	65	139	24	23	13	50	50	23	39	50	74	23	52	50	24	73	13	89	7
Liu (14)	2020	N	366	67	299	Microarray	–	–	–	–	24	8	92	56	35	8	151	56	59	8	243	56	24	43	92	207	7
Fang (33)	2021	N	1242	568	674	FQ-PCR	330	806	519	829	55	293	103	258	220	293	313	258	275	293	416	258	55	513	103	571	8
Wang (35)	2021	N	250	130	120	FQ-PCR	160	100	64	176	55	25	18	74	50	25	28	74	105	25	46	74	55	75	18	102	7
Niu (34)	2021	N	198	83	115	Sequencing	70	96	65	165	21	34	9	59	28	34	47	59	49	34	56	59	21	62	9	106	7
Zhang (38)	2022	N	200	100	100	Sequencing	97	103	67	133	26	29	12	45	45	29	43	45	71	29	55	45	26	74	12	88	7
Lu (36)	2022	S	1251	345	906	TaqMan	–	–	–	–	37	179	86	444	129	179	376	444	166	179	462	444	37	308	86	820	8
Mo (37)	2022	S	180	80	100	FQ-PCR	122	38	77	123	52	10	15	38	18	10	47	38	70	10	62	38	52	28	15	85	7
Gu (39)	2023	S	5614	1054	4560	FQ-PCR	–	–	–	–	85	568	369	2415	401	568	1776	2415	486	568	2145	2415	85	969	369	4191	8
Huang (40)	2023	S	822	146	676	Sequencing	–	–	–	–	29	76	53	404	41	76	219	404	70	76	272	404	29	117	53	623	8
Liu (41)	2023	N	92	14	78	TaqMan	–	–	–	–	7	4	14	31	3	4	33	31	10	4	47	31	7	7	14	64	6

GDM, gestational diabetes mellitus; QF-PCR, quantitative fluorescent polymerase chain reaction; FQ-PCR, fluorescence quantitative polymerase chain reaction; NOS, Newcastle-Ottawa Scale.

### The association between the *MTHFR* gene C677T polymorphism and GDM risk in the Chinese population

**Figure 2:**
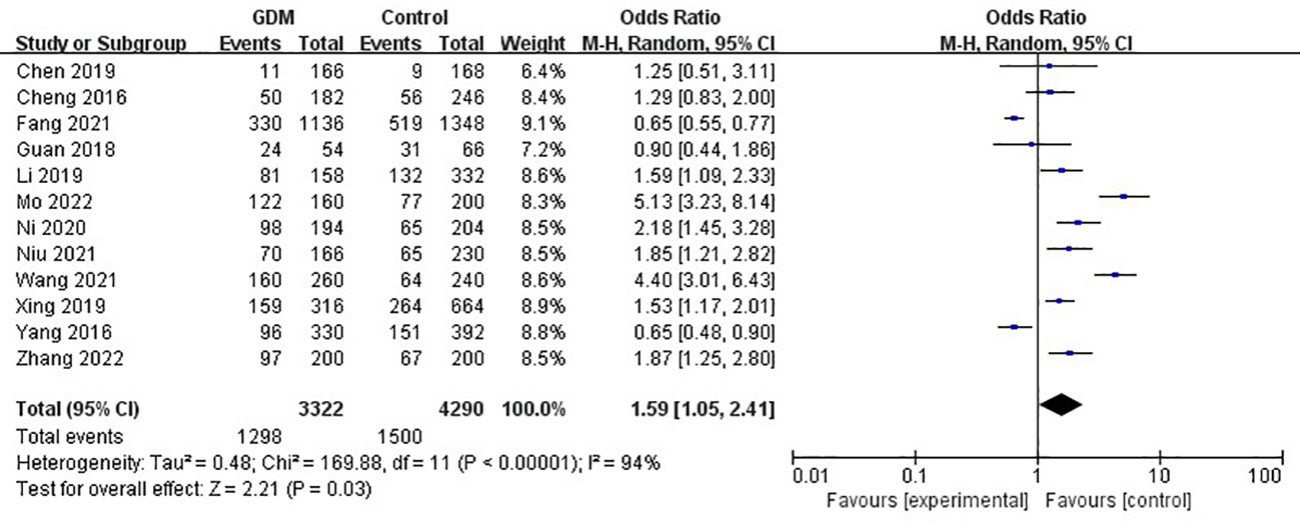
The association between *MTHFR* gene C677T polymorphism and risk of gestational diabetes mellitus in Chinese population under the allele model.

**Figure 3:**
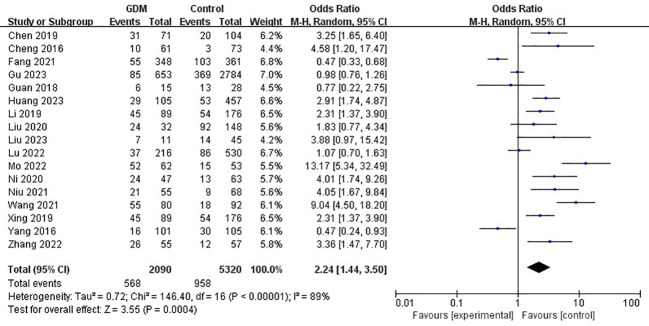
The association between *MTHFR* gene C677T polymorphism and risk of gestational diabetes mellitus in Chinese population under the homozygote model.

**Figure 4:**
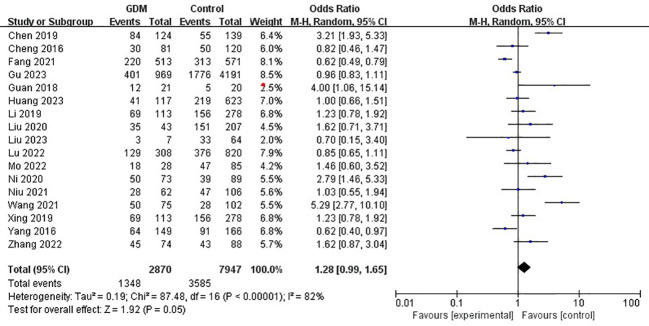
The association between *MTHFR* gene C677T polymorphism and risk of gestational diabetes mellitus in Chinese population under the heterozygote model.

**Figure 5:**
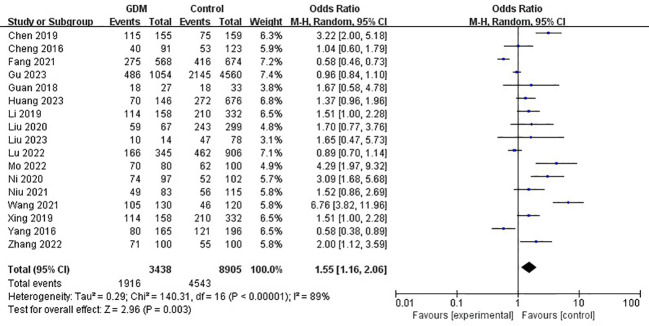
The association between *MTHFR* gene C677T polymorphism and risk of gestational diabetes mellitus in Chinese population under the dominant model.

**Figure 6:**
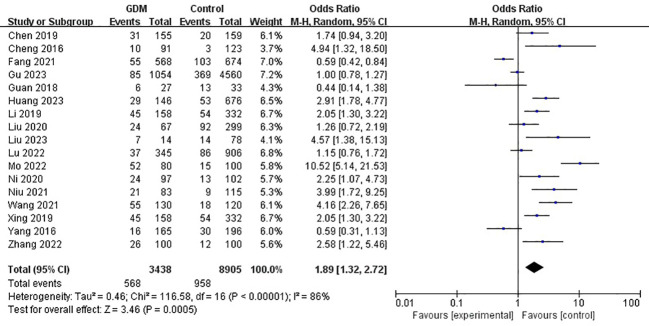
The association between *MTHFR* gene C677T polymorphism and risk of gestational diabetes mellitus in Chinese population under the recessive model.

The pooled results demonstrated that the *MTHFR* gene C677T polymorphism was significantly related to the risk of GDM in the allele model (OR=1.59, 95%:1.05-2.41, P=0.03; I^2^ = 94%, P<0.001) (**Figure 2**), homozygote model (OR=2.24, 95%:1.44-3.50, P<0.001; I^2^ = 89%, P<0.001) (**Figure 3**), heterozygote model (OR=1.28, 95%:0.99-1.65, P=0.05; I^2^ = 82%, P<0.001) (**Figure 4**), dominant model (OR=1.55, 95%:1.16-2.06, P=0.003; I^2^ = 89%, P<0.001) (**Figure 5**) and recessive model (OR=1.89, 95%:1.32-2.72, P<0.001; I^2^ = 86%, P<0.001) (**Figure 6**). Patients with the T variant allele were more likely to develop GDM (**Table 2**).

**Table 2:** Results of meta-analysis.

	No. of studies	Odds ratio	95% confidence interval	P value	I^2^(%)	P value
Allele model (T vs C)	12	1.59	1.05-2.41	0.03	94	<0.001
Region						
North	6	1.57	0.81-3.04	0.19	95	<0.001
South	6	1.62	0.92-2.85	0.09	91	<0.001
Homozygote model (TT vs CC)	17	2.24	1.44-3.50	<0.001	89	<0.001
Region						
North	8	2.26	0.96-5.31	0.06	91	<0.001
South	9	2.22	1.28-3.83	0.004	88	<0.001
Heterozygote model (TC vs CC)	17	1.28	0.99-1.65	0.05	82	<0.001
Region						
North	8	1.50	0.85-2.65	0.16	86	<0.001
South	9	1.17	0.88-1.56	0.28	79	<0.001
Dominant model (TT+TC vs CC)	17	1.55	1.16-2.06	0.003	89	<0.001
Region						
North	8	1.72	0.91-3.27	0.10	91	<0.001
South	9	1.43	1.03-1.98	0.03	87	<0.001
Recessive model (TT vs TC+CC)	17	1.89	1.32-2.72	<0.001	86	<0.001
Region						
North	8	1.82	0.97-3.38	0.06	87	<0.001
South	9	1.97	1.22-3.19	0.006	87	<0.001

However, subgroup analysis stratified by region revealed a significant association between the *MTHFR* gene C677T polymorphism and an increased risk of GDM only among the southern population in the allele model (OR=1.62, 95%:0.92-2.85, P=0.09; I^2^ = 91%, P<0.001), homozygote model (OR=2.22, 95%:1.28-3.83 P=0.004; I^2^ = 88%, P<0.001), heterozygote model (OR=1.17, 95%:0.88-1.56, P=0.28; I^2^ = 79%, P<0.001), dominant model (OR=1.43, 95%:1.03-1.98, P=0.03; I^2^ = 87%, P<0.001) and recessive model (OR=1.97, 95%:1.22-3.19, P=0.006; I^2^ = 87%, P<0.001). Although a significant difference in the allele model and heterozygote model was not shown, an obvious tendency was observed (**Table 2**). Thus, southern Chinese pregnant women with the T variant allele of the *MTHFR* gene C677T polymorphism showed significantly increased susceptibility to GDM overall.

### Sensitivity analysis

Sensitivity analysis for the five gene models was performed. Overall, the results in the homozygote model (**Supplementary Figure 1B**), dominant model (**Supplementary Figure 1D**) and recessive model (**Supplementary Figure 1E**) were stable and reliable. However, studies by Mo et al. (37) and Wang et al. (35) showed an impact on the overall results in the allele model (**Supplementary Figure 1A**), and several studies (14, 28–30, 32, 37, 39, 40 31, 35, 38) showed an impact on the overall results in the heterozygote model (**Supplementary Figure 1C**). Thus, more high-quality studies are still needed to further confirm our findings.

### Publication bias

Similarly, publication bias for the five gene models was assessed. According to Begg’s funnel plots (**Supplementary Figures 2A–E**) and Egger’s test P values (allele model: P=0.045; homozygote model: P=0.011; heterozygote model: P=0.037; dominant model: P=0.010; recessive model: P=0.028), significant publication bias was observed. Thus, the fill-and-trim method was applied to identify potentially unpublished studies. According to filled funnel plots, no potentially unpublished publications for the allele model (**Supplementary Figure 3A**) or heterozygote model (**Supplementary Figure 3C**) were found. Three, six and two potentially unpublished studies were detected for the homozygote model (**Supplementary Figure 3B**), dominant model (**Supplementary Figure 3D**) and recessive model (**Supplementary Figure 3E**), respectively. The potentially unpublished studies had no significant effect on the overall results in the homozygote model (filled OR=1.74, 95% CI: 1.12-2.68, P=0.013) and recessive model (filled OR=1.63, 95% CI: 1.13-2.36, P=0.009). However, in the dominant model, a significant effect on the overall results caused by the six potentially unpublished studies was observed (filled OR=1.06, 95% CI: 0.80-1.42, P=0.678). Therefore, to further clarify the association between the *MTHFR* gene C677T polymorphism and the risk of GDM, more high-quality studies should be performed.

### Trial sequential analysis

**Supplementary Figure 4** shows the TSA results. In **Supplementary Figures 4B** and **E**, the cumulative Z-curve cut crossed TSA boundaries (red polylines), indicating that the information size was adequate and that the conclusions were robust. However, the **Supplementary Figures 4A, C** and **D** show negative results. Therefore, more case-control studies are still needed to further confirm our findings.

## Discussion

The current meta-analysis included 17 relevant studies with 12345 participants and explored the relationship between the *MTHFR* gene C677T polymorphism and susceptibility to GDM among pregnant Chinese women. Based on currently available evidence, we demonstrated that the T variant allele could serve as a risk factor for GDM among the southern Chinese population. However, due to the limitations in the included studies and our analysis, more randomized controlled trials (RCTs) are needed to further confirm our findings.

It has been reported that the serum Hcy level in GDM patients is significantly higher than that in non-GDM patients (34). Serum Hcy levels have been identified as an independent risk factor for the occurrence of GDM, indicating a strong correlation between elevated Hcy levels and the development of GDM. Hcy can serve as a marker for monitoring the progression of GDM and as a potential predictive factor for evaluating microvascular changes, reflecting the status of blood sugar control (34). A possible reason is that during pregnancy, the maternal body and placenta secrete anti-insulin hormones, leading to increased insulin resistance, which in turn causes elevated Hcy levels. Elevated Hcy levels further inhibit pancreatic β-cell insulin secretion, resulting in an increase in blood glucose levels (42). These two factors have a reciprocal causal relationship and mutually influence each other. Therefore, elevated Hcy levels may serve as a pathological basis for promoting the occurrence and development of GDM (42).

The reduction in enzyme activity caused by the C677T mutation in the *MTHFR* gene is an important factor leading to elevated Hcy levels (43). *MTHFR* is a key enzyme involved in Hcy metabolism, catalyzing the conversion of 5,10-methylenetetrahydrofolate to 5-methyltetrahydrofolate, which promotes the conversion of Hcy to methionine (14). The C677T mutation site can lead to the substitution of methionine for alanine in *MTHFR* isoleucine, resulting in decreased *MTHFR* enzyme activity. This impedes the conversion of Hcy to methionine, leading to the accumulation of Hcy in the body and subsequently causing an increase in the serum Hcy concentration, which contributes to the development of GDM (44). The results from the study by Pawlik A et al. showed that *MTHFR* CT heterozygous mutations reduced enzyme activity to approximately 75% of the CC type, while TT genotype mutations led to even more significant decreases, reaching approximately 50% or lower (45). The aforementioned reports confirm that different genotypic mutations of *MTHFR* C677T can cause a reduction in *MTHFR* enzyme activity, resulting in elevated serum Hcy levels. Therefore, the T variant allele in the *MTHFR* gene C677T polymorphism leads to an increased risk of GDM.

Most included studies revealed a relationship between the *MTHFR* gene C677T polymorphism and an increased risk of GDM. However, studies by Fang et al. (33) and Yang et al. (27) reported opposite results, and they suggested several potential causes. Yang et al. did not find a significant correlation between mutations of *MTHFR* C677T and plasma Hcy levels, suggesting that the T variant allele in the *MTHFR* gene C677T polymorphism may have a relatively minor impact on Hcy metabolism in pregnant Han Chinese women. However, this mutation may reduce the risk of GDM through the alteration of other metabolic pathways within the body, thereby masking the potential adverse effects of Hcy metabolism changes caused by the C-T mutation in *MTHFR* C677T (27). In addition, the metabolism of Hcy is regulated by various factors, including genetics and nutrition. The potential influence of confounding factors such as plasma/red blood cell folate levels and the glomerular filtration rate in the study subjects might lead to biases in the research findings (27). Moreover, the sample size with 363 patients in the study by Yang et al. was relatively small, and the test performance of their results was only 0.48.

There are several limitations in our meta-analysis. First, the sample sizes in several included studies were relatively small, which might cause some bias. Second, the genotyping methods in the included studies differed, and the accuracy of different methods may vary. Third, a more detailed subgroup analysis based on other important parameters, such as race and age, could not be performed because the original data were unavailable. Fourth, significant heterogeneity among the included studies was detected.

## Conclusion

The *MTHFR* gene C677T polymorphism is closely related to susceptibility to GDM in the southern Chinese population, and the C-T mutation serves as an important genetic risk factor for GDM.

## Data availability statement

The original contributions presented in the study are included in the article/**Supplementary Material**. Further inquiries can be directed to the corresponding author.

## Author contributions

XT: Conceptualization, Data curation, Formal Analysis, Methodology, Writing – original draft. HC: Project administration, Supervision, Validation, Writing – review & editing.
