# Supplementary material for: Association between MTHFR gene C677T polymorphism and gestational diabetes mellitus in Chinese population: a meta-analysis
Source: Front Endocrinol (Lausanne). 2023 Oct 30;14:1273218. doi: 10.3389/fendo.2023.1273218 (PMC10642752; doi:10.3389/fendo.2023.1273218)

Supplementary figure 2A. Begg's funnel plot for the association between *MTHFR* gene C677T polymorphism and risk of gestational diabetes mellitus in Chinese population under the allele model (T vs C).

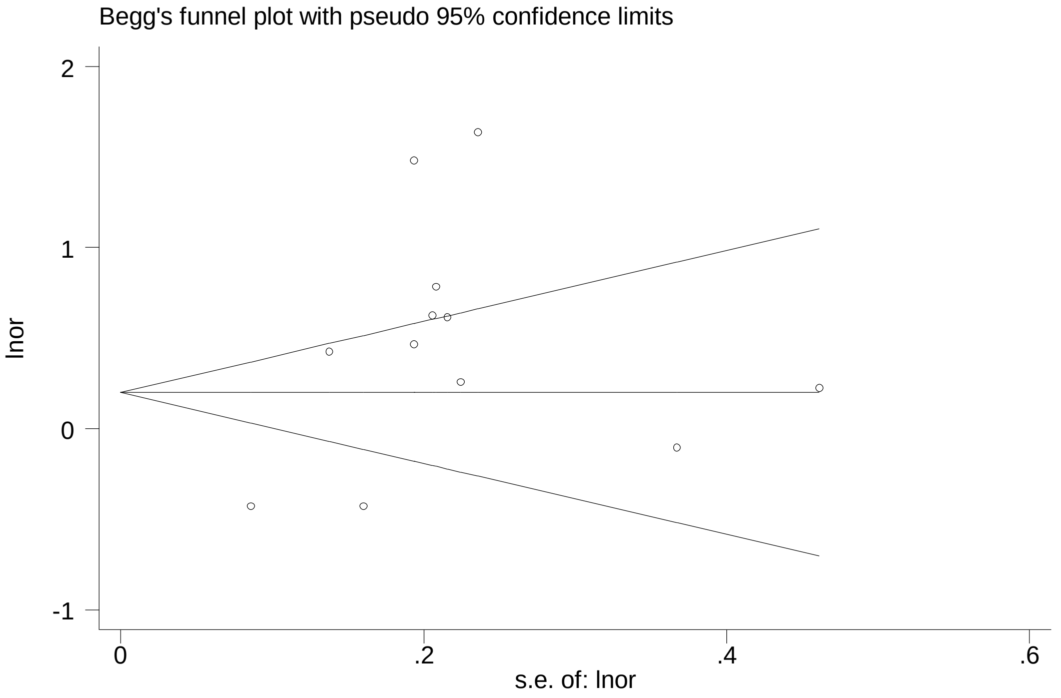

Supplementary figure 2B. Begg's funnel plot for the association between *MTHFR* gene C677T polymorphism and risk of gestational diabetes mellitus in Chinese population under the homozygote model (TT vs CC).

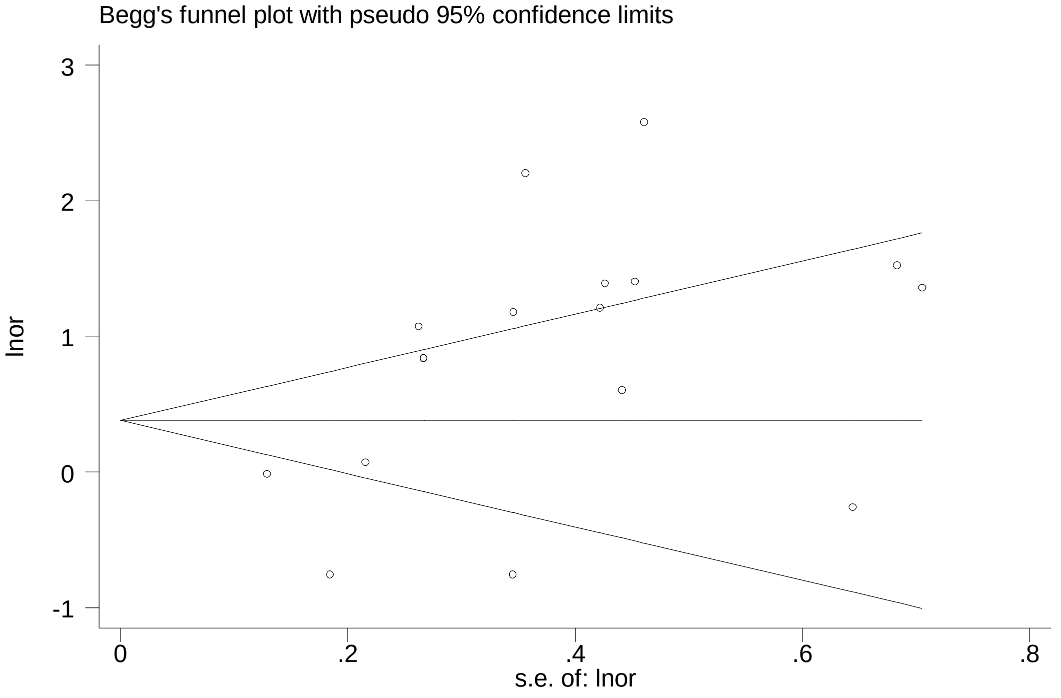

Supplementary figure 2C. Begg's funnel plot for the association between *MTHFR* gene C677T polymorphism and risk of gestational diabetes mellitus in Chinese population under the heterozygote model (TC vs CC).

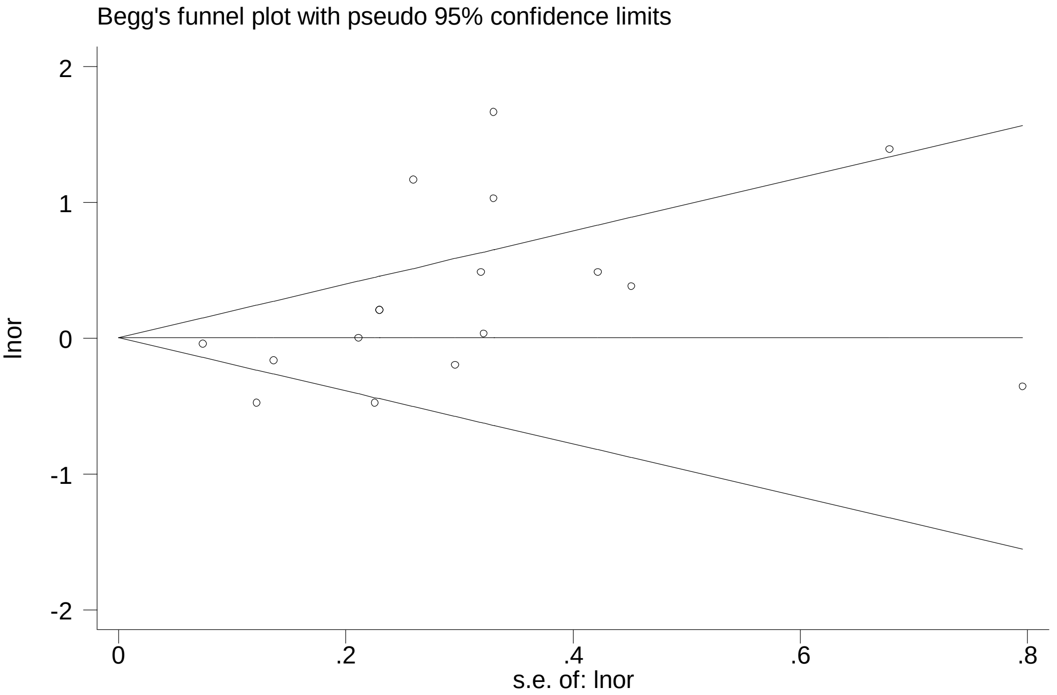

Supplementary figure 2D. Begg's funnel plot for the association between *MTHFR* gene C677T polymorphism and risk of gestational diabetes mellitus in Chinese population under the dominant model (TT+TC vs CC).

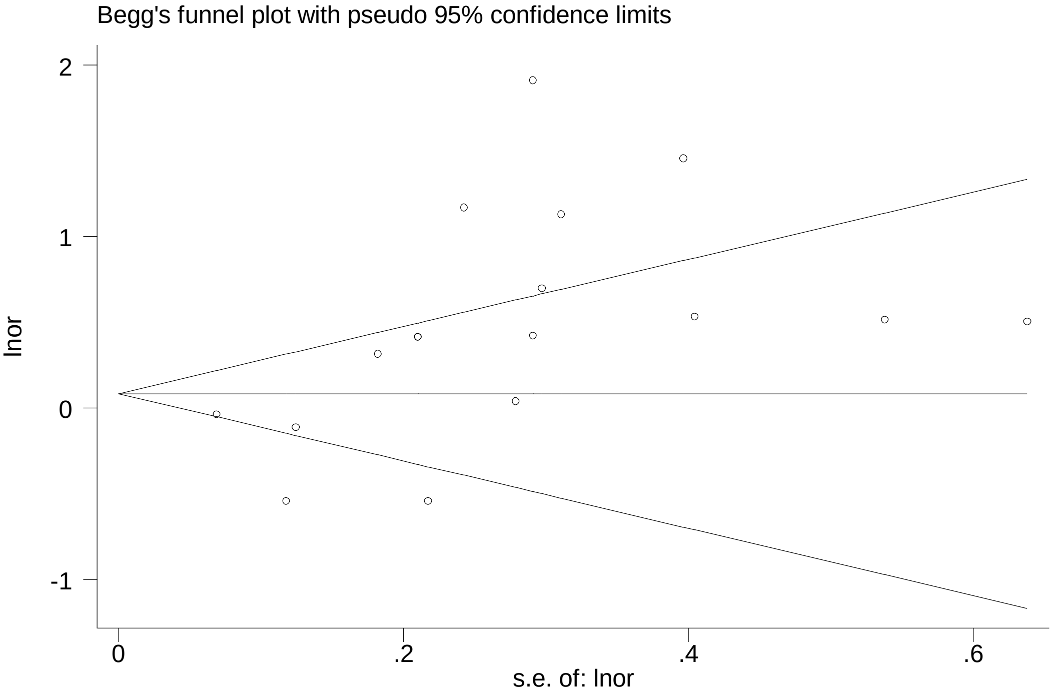

Supplementary figure 2E. Begg's funnel plot for the association between *MTHFR* gene C677T polymorphism and risk of gestational diabetes mellitus in Chinese population under the recessive model (TT vs TC+CC).

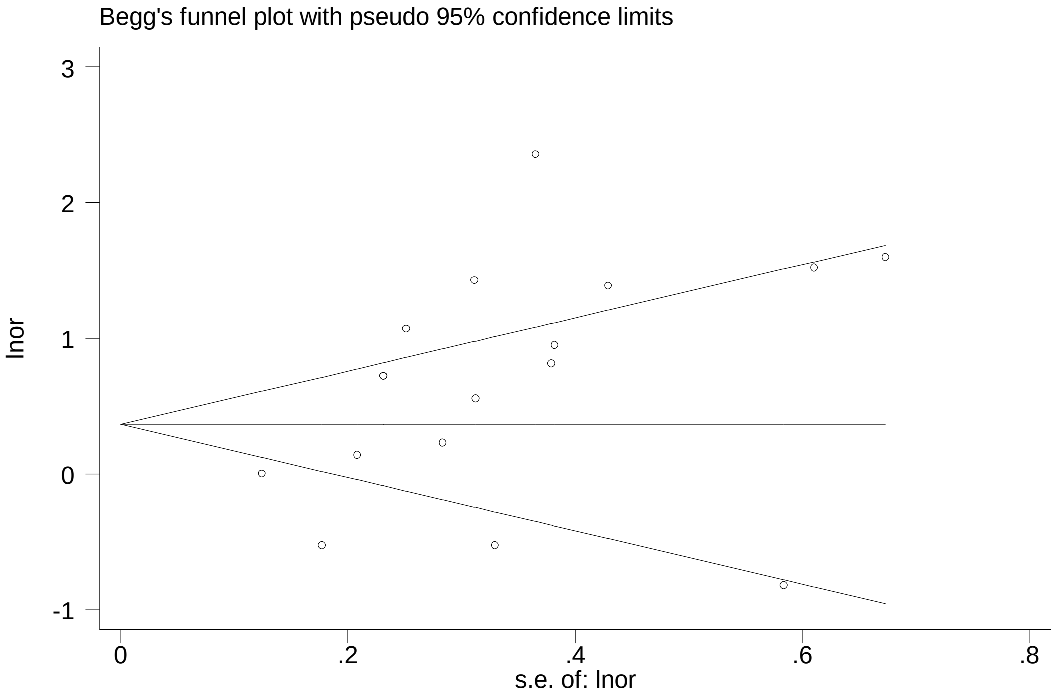

Supplement: Supplementary file 2 [file DataSheet_2.pdf]
