# Supplementary material for: Association between MTHFR gene C677T polymorphism and gestational diabetes mellitus in Chinese population: a meta-analysis
Source: Front Endocrinol (Lausanne). 2023 Oct 30;14:1273218. doi: 10.3389/fendo.2023.1273218 (PMC10642752; doi:10.3389/fendo.2023.1273218)

Supplementary figure 4A. Trial sequential analysis plot for the association between *MTHFR* gene C677T polymorphism and risk of gestational diabetes mellitus in Chinese population under the allele model.

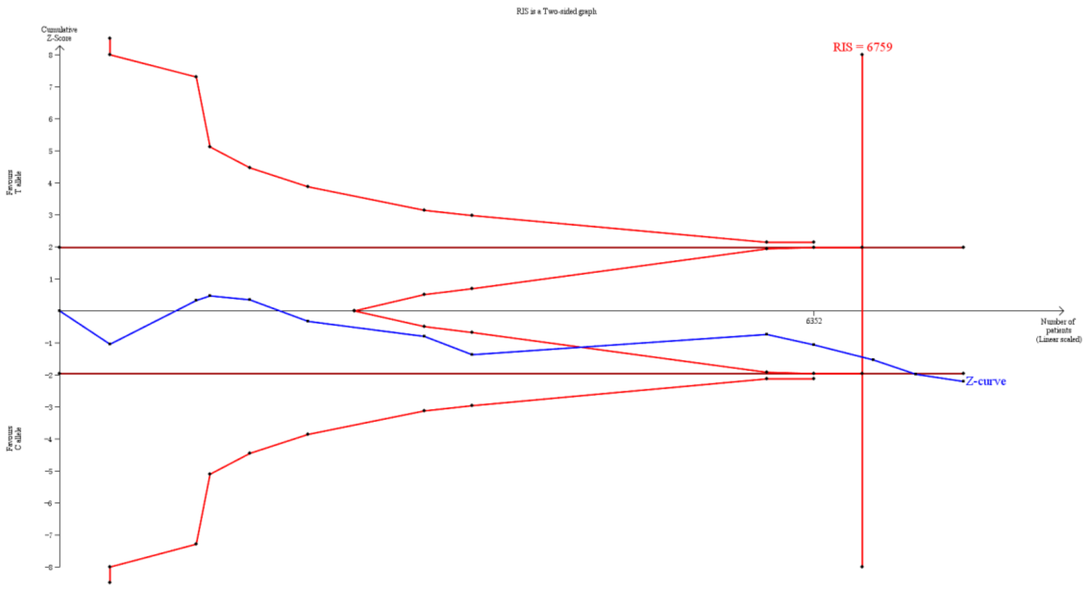

Supplementary figure 4B. Trial sequential analysis plot for the association between *MTHFR* gene C677T polymorphism and risk of gestational diabetes mellitus in Chinese population under the homozygote model.

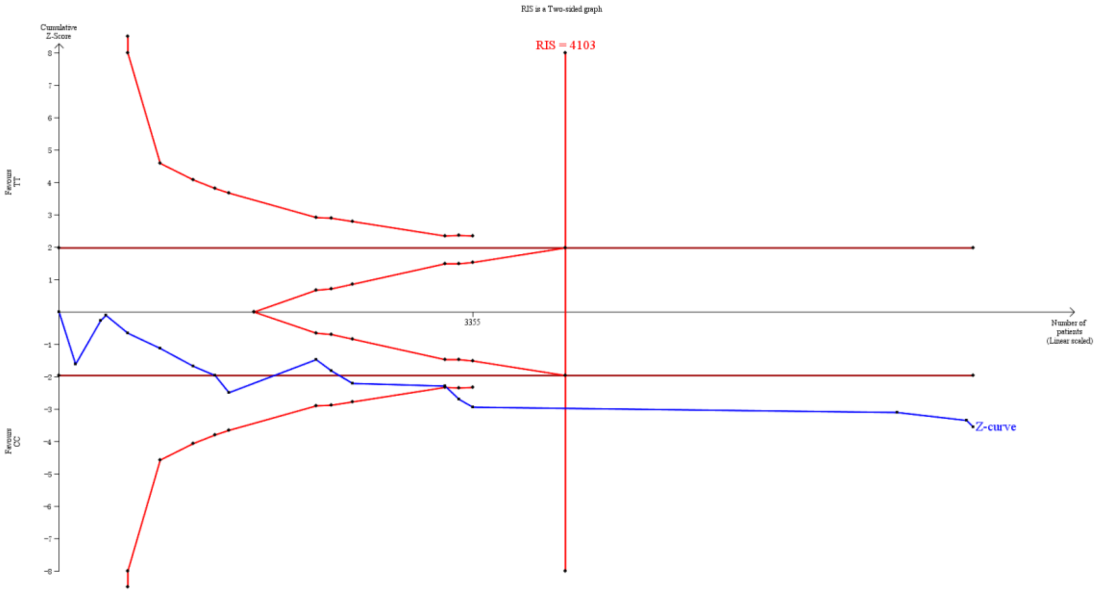

Supplementary figure 4C. Trial sequential analysis plot for the association between *MTHFR* gene C677T polymorphism and risk of gestational diabetes mellitus in Chinese population under the heterozygote model.

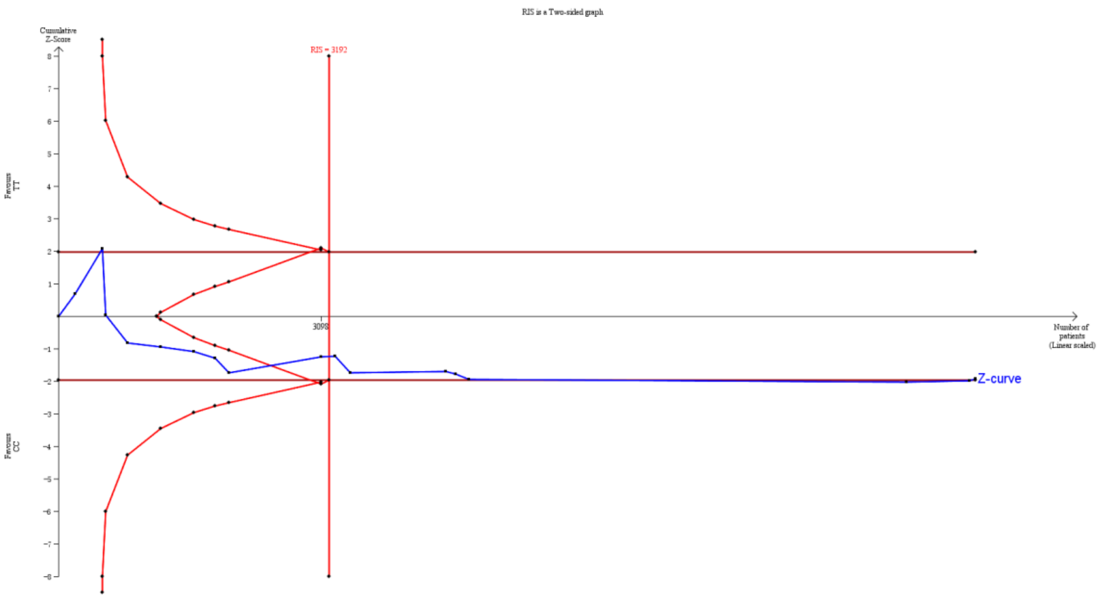

Supplementary figure 4D. Trial sequential analysis plot for the association between *MTHFR* gene C677T polymorphism and risk of gestational diabetes mellitus in Chinese population under the dominant model.

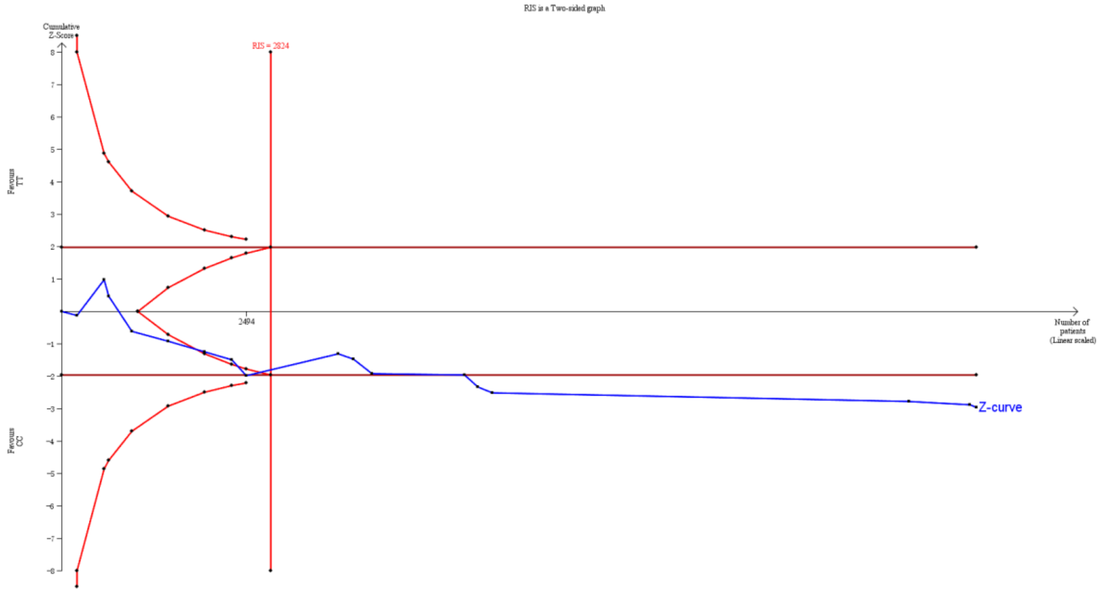

Supplementary figure 4E. Trial sequential analysis plot for the association between *MTHFR* gene C677T polymorphism and risk of gestational diabetes mellitus in Chinese population under the recessive model.

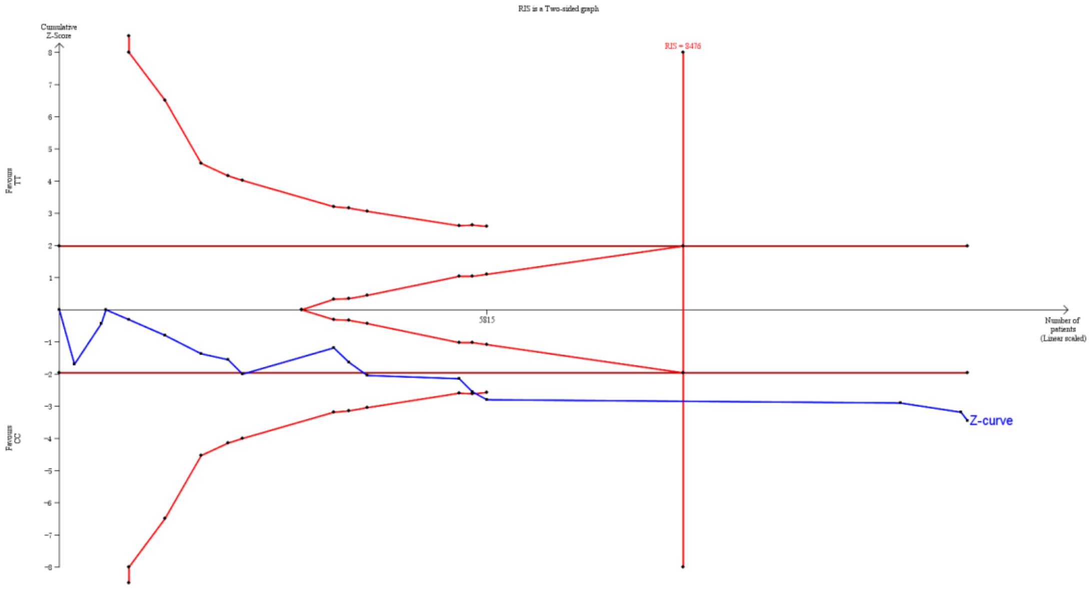

Supplement: Supplementary file 4 [file DataSheet_4.pdf]
