# Supplementary material for: Association between MTHFR gene C677T polymorphism and gestational diabetes mellitus in Chinese population: a meta-analysis
Source: Front Endocrinol (Lausanne). 2023 Oct 30;14:1273218. doi: 10.3389/fendo.2023.1273218 (PMC10642752; doi:10.3389/fendo.2023.1273218)
Supplement: Supplementary file 5 [file Table_1.docx]

Supplementary table 1. Detailed results of NOS score.

| Author | Selection | | | | Comparability | Outcome measurement | | | Score |
| --- | --- | --- | --- | --- | --- | --- | --- | --- | --- |
|  | Representativeness of the exposed cohort | Selection of the non-exposed cohort | Ascertainment of exposure | Outcome of interest | Comparability of cohorts | Assessment of outcome | Time of follow-up | Adequacy of follow-up |  |
| Cheng (Cheng et al., 2016) | 1 | 1 | 1 | 1 | 0 | 1 | 1 | 1 | 7 |
| Yang (Yang, 2016) | 1 | 1 | 1 | 1 | 0 | 1 | 1 | 1 | 7 |
| Guan (Guan & Yu, 2018) | 0 | 1 | 1 | 1 | 0 | 1 | 1 | 1 | 6 |
| Li (S. Li et al., 2019) | 1 | 1 | 1 | 1 | 1 | 1 | 1 | 1 | 8 |
| Xing (Xing et al., 2019) | 1 | 1 | 1 | 1 | 1 | 1 | 1 | 1 | 8 |
| Chen (Yugang Chen et al., 2019) | 1 | 1 | 1 | 1 | 0 | 1 | 1 | 1 | 7 |
| Ni (Ni et al., 2020) | 1 | 1 | 1 | 1 | 0 | 1 | 1 | 1 | 7 |
| Liu (P. J. Liu et al., 2020a) | 1 | 1 | 1 | 1 | 0 | 1 | 1 | 1 | 7 |
| Fang (Fang et al., 2021) | 1 | 1 | 1 | 1 | 1 | 1 | 1 | 1 | 8 |
| Wang (L. Wang et al., 2021) | 1 | 1 | 1 | 1 | 0 | 1 | 1 | 1 | 7 |
| Niu (Niu et al., 2021) | 1 | 1 | 1 | 1 | 0 | 1 | 1 | 1 | 7 |
| Zhang (Zhang, 2022) | 1 | 1 | 1 | 1 | 0 | 1 | 1 | 1 | 7 |
| Lu (Lu & Liang, 2022) | 1 | 1 | 1 | 1 | 1 | 1 | 1 | 1 | 8 |
| Mo (Mo et al., 2022) | 1 | 1 | 1 | 1 | 0 | 1 | 1 | 1 | 7 |
| Gu (Gu et al., 2023) | 1 | 1 | 1 | 1 | 1 | 1 | 1 | 1 | 8 |
| Huang (Huang et al., 2023) | 1 | 1 | 1 | 1 | 1 | 1 | 1 | 1 | 8 |
| Liu (H.-Y. Liu et al., 2023) | 0 | 1 | 1 | 1 | 0 | 1 | 1 | 1 | 6 |

NOS: Newcastle-Ottawa Scale.
